# The Influence of Mental, Physical, and Social Activity on Episodic Memory of Americans Aged 50 and Older

**DOI:** 10.1093/geroni/igaa057.3298

**Published:** 2020-12-16

**Authors:** Ayse Malatyali, Shirley Gordon, John Morris, Lisa Wiese, Christine Williams

**Affiliations:** Florida Atlantic University, Boca Raton, Florida, United States

## Abstract

The aim of this study was to describe the relationship between mental, physical, and social activity and episodic memory (EM) and further examine whether the relationship between these activities and EM is moderated by each activity type and demographics such as gender, marital status, and race. Participants were 3,903 cognitively intact persons who completed the 2016 Health and Retirement Study core interview and the 2015 Consumptions and Activities Mail Survey. Preliminary results showed no significant relationship between EM and mental, physical, and social activity. Mental activity significantly moderated the relationship between physical activity and EM, revealing a positive association between physical activity and EM for participants reporting low mental activity levels and a negative but non-significant association for participants with high mental activity scores. Although women demonstrated higher EM scores than men, there was a negative association between mental activity and EM for women. A significant reduction of EM performance was observed in participants who were divorced and widowed, compared to participants who were married. There was a positive relationship between physical activity and EM only for participants who were widowed. Furthermore, race significantly related to EM: African Americans had lower EM scores than Whites/Caucasians, but no significant association was found for the other race groups. Effect sizes were small throughout the analyses. Results emphasize the underlying relationship between different activity types and demographics in terms of EM performance. Further investigation is needed to explore changes over time in this relationship for persons who are cognitively intact or impaired.

